# A chromosome-level genome assembly of the Echiura *Urechis unicinctus*

**DOI:** 10.1038/s41597-023-02885-7

**Published:** 2024-01-18

**Authors:** Yunying Cheng, Ruanni Chen, Jinlin Chen, Wanlong Huang, Jianming Chen

**Affiliations:** 1 Fujian Key Laboratory on Conservation and Sustainable Utilization of Marine Biodiversity, Fuzhou Institute of Oceanography, College of Geography and Oceanography, Minjiang University, Fuzhou, 350108 China; 2 Novogene Bioinformatics Institute, Beijing, China

**Keywords:** Genomics, Sequencing

## Abstract

Echiura is a distinctive family of unsegmented sausage-shaped marine worms whose phylogenetic relationship still needs strong evidence from the phylogenomic analysis. In this family, *Urechis unicinctus* is known for its high nutritional and medicinal value and adaptation to harsh intertidal conditions. Herein, we combined PacBio long-read, short-read Illumina and Hi-C sequencing, generating a high-quality chromosome-level genome assembly of *U. unicinctus*. The assembled genome spans ~1,138.6 Mb with a scaffold N50 of 68.3 Mb, of which 1,113.8 Mb (97.82%) were anchored into 17 pseudo-chromosomes. The BUSCO analysis demonstrated the completeness of the genome assembly and gene model prediction are 93.5% and 91.5%, respectively. A total of 482.1 Mb repetitive sequences, 21,524 protein-coding genes, 1,535 miRNAs, 3,431 tRNAs, 124 rRNAs, and 348 snRNAs were annotated. This study significantly improves the quality of *U. unicinctus* genome assembly, sets the footsteps for molecular breeding and further study in genome evolution, genetic and molecular biology of *U. unicinctus*.

## Background & Summary

Echiura, commonly referred to as spoon worms, are bilaterally symmetrical and coelomate marine invertebrates with a sausage-shaped body living in burrows in the sediments. They possess annelid-like morphological features, including the ladder-like nervous system, the ultrastructure of cuticle and chaetae, and the larval nervous system segments^1^, however, they have secondarily lost segmentation as adults, providing a particularly important model for understanding the mechanism underlying segment formation and secondary loss^2^. Furthermore, the evolutionary relationship between the Echiura and Annelida is in a long- standing controversy. Given the lack of segmentation as adults, Echiura has generally been regarded as a separate phyla closely related to Annelida. Recently, some researchers advised the inclusion of Echiura into Annelida based on the increasing amounts of morphological, molecular phylogenetic and phylogenomic evidence, including expressed sequence tags, transcriptome, and mitochondrial genome^3–8^. As solid evidence for a better understanding of their deep-level evolutionary relationships, phylogenomic analysis from high-quality chromosome-level genome data of the Echiura is still lacking.

*Urechis unicinctus* (also called the penis fish or the fat innkeeper worm), belonging to the Echiura, is a deposit-feeding burrowing organism that inhabits the intertidal zones along the Korean and Japanese coast and Bohai Gulf on the northeast coast of China. The intertidal zones are peculiar and dynamic areas which are vulnerable to a host of stressors, like steep gradients in temperature and oxygen concentration, threats from pathogen infections, pollution, and toxic substances^9^. The *U. unicinctuss* without adaptive immunity can survive in such harsh environments, providing an exciting resource for investigating environmental adaptative evolution. In addition, this endemic Echiuran species has essential ecological and socioeconomic significance and has become an important cultured aquaculture species due to its desirable flavour, nutrient-rich and high medicinal values in Asian countries, especially in China, Japan, and Korea^10^. The first draft genome assembly of *U. unicinctuss* based on Illumina short reads was published in 2021^11^. However, due to the limitations of the sequencing technique and assembly algorithm, the genome assembly with a contig N50 length of 0.458 kb and scaffold N50 length of 0.517 kb remains highly fragmented (Table 1), which lags far behind the demand for further study of genetic and molecular in *U. unicinctuss*. Hence, a high-quality chromosome-scale genome assembly of *U. unicinctus*s are essential in elucidating its genome evolution and adaptive evolution, and providing theoretical support for the species’ culture.

**Table 1:** Comparative statistics of *U. unicinctus* genome assemblies in 2021 and 2023.

Year	2021	2023
Sequencing libraries	Illumina	Illumina + 10 × genomics + PacBio + Hi-C
Bases (Gbp)	75.3	97.74 (Illumina) + 107.42 (10 × genomics) + 142.1 (Pacbio) + 159.47 (Hi-C)
Insert size (bp)	250–300	350 (Illumina) + 350 (10 × genomics) + 20 Kb(Pacbio) + 350 (Hi-C)
Coverage	57.137	346 × (Illumina) + 89.52 × (10 × genomics) + 118.42 × (Pacbio) + 132.8 9 × (Hi-C)
RNA (Gbp)	—	26.51
Chromosomes	—	17
Genome size (Mbp)	1,098.7	1,146.9
Contigs	3,742,050	7,429
Contig N50 (Kbp)	0.458	525.71
Scaffolds	3,551,270	1,394
Scaffold N50 (Kbp)	0.517	69,985.25
GC content (%)	40.28	39.38

**Fig. 1:**
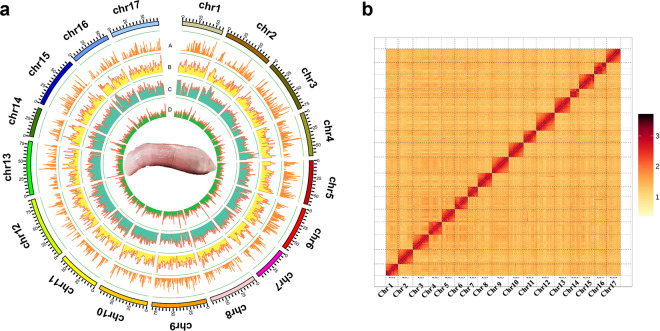
Overview of *U. unicinctus* genome. (**a**) Circos plot of genomic features. From outer to inner ring: (A) gene density; (B) GC content; (C) transposable element abundance; (D) Tandem repeat density. Insert: image of adult *U. unicinctus*. (**b**) Pseudochromosomes interaction heatmap of *U. unicinctus* based on Hi-C assembly. Color block indicates the intensity of interaction from yellow (low) to red (high).

Here, we present a high-quality chromosome-scale genome assembly of *U. unicinctus* obtained by combining Illumina, PacBio, and high-throughput chromosome conformation capture (Hi-C) sequencing technology toolkits. The *U. unicinctus* genome, with a total size of ~1,138.6 Mb, was assembled into 1,394 scaffolds (N50 = 68.3 Mb). A total of 1,113.8 Mb assembled sequences (97.82%) were further anchored to 17 pseudochromosomes (Fig. 1). The quality of the genome assembly is significantly higher than that of the previously published version (NCBI accession No. PRJNA603659), with contig N50 being ~1,160 times higher and scaffold N50 being ~135,472 times higher (Table 1)^11^. The completeness, accuracy, and contiguity of the genome assembly were evaluated by Benchmarking Universal Single-Copy Ortholog (BUSCO) analysis, Core Eukaryotic Genes Mapping Approach (CEGMA), re-alignment between clean Illumina reads and the genome assembly, and SNP identification. Of the assembled genome, 482.1 Mb (42.34%) were repetitive sequences with a dominance of DNA elements. Additionally, a total of 21,524 protein-coding genes were annotated, of which 99.5% could be functionally annotated. This chromosome-level genome assembly builds the foundation for the understanding of genome evolution and evolutionary adaption and provides a valuable tool for further studies on the genetic and molecular biology of *U. unicinctus*.

## Methods

### Samples collection and whole-genome sequencing

Adult *U. unicinctus* samples were obtained from the field of Xiyan, Yantai, Shandong Province, China (121°25’E, 37°56’N), and genomic DNA extracted from the muscle tissue was collected for whole-genome sequencing using a QIAGEN DNeasy Blood & TissueKit (QIAGEN, Shanghai, China). Paired-end Illumina sequence library with insert size 350 bp and 10× Genomics linked-read library were sequenced by Illumina HiSeq X Ten platform with 97.74 Gb of short- read sequencing data (Table 1). For long-read sequencing, a library with an insert of 20 kb was constructed using SMRTbell Template Prep Kits, followed by PacBio single-molecule real- time (SMRT) sequencing using Pacbio Sequel Platform (Pacific Biosciences, Menlo Park, USA), generating approximately 142.1 Gb of long-read raw data.

### Transcriptome sequencing

For transcriptome sequencing, four tissues, including intestines, gonads, blood, and muscle, were sampled from the same individual and stored in liquid nitrogen. RNA was extracted from these tissues and used for transcriptome sequencing, respectively. The cDNA paired- end libraries were prepared and sequenced on an Illumina HiSeq X sequencer (Paired-end 350 bp reads). Approximately 26.51 Gb of clean data were yielded from the RNA-seq raw data after quality control using fastp v. 0.21.0^12^ (Table 1).

### Genome size estimation and assembly

Jellyfish v. 2.1.3 method^13^ with k-mer distribution was employed to calculate k-mer frequency (*k* = 17) based on the high-quality paired-end reads (with an insert size of 350 bp). The distribution of 17-mer depends on the characteristic of the genome and follows a Poisson’s distribution. The genome size was estimated to be 1,396.33 MB with K-mer depth of 58. The genome heterozygosity and repeat ratio are 1.25% and 53.86%, respectively (Table 2).

**Table 2:** The genome size estimation of *U. unicinctus* by k-mer distribution.

K-mer	K-mer number	K-mer Depth	Genome Size	Revised Genome Size (Mbp)	Heterozygous Ratio(%)	Repeat (%)
17	82,177,594,152	58	1,416.86	1,396.33	1.25	53.86

The WTDBG software v. 2.5, https://github.com/ruanjue/wtdbg) was used to assemble the contig of the *U. unicinctus* genome with parameters setting as ‘--node-drop 0.20 --node-len 2304 --node-max 150 -s 0.05 -e 3′. Then, Racon v. 1.3.1^14^ with default parameters was used to correct errors of contigs assembly by PacBio data. The resulting contigs were connected to super-scaffolds by 10× Genomics linked-read data using the fragScaff software v. 140324 with parameters setting as ‘-maxCore 200 -m 3000 -q 30 -C 5’^15^. Lastly, pilon v. 1.22 with parameters setting as ‘-Xmx300G --diploid --threads 20’^16^ was used to perform the second round of error correction with short paired-end reads generated from Illumina Hiseq X Ten Platforms. The total length of the contig assembly was 1130.4 Mb with the contig N50 size of 528.1 Kb (Table 3). For the scaffolding step, SSPACE v. 3.0^17^ was first used to construct scaffolds using HiSeq data from all the mate-pair libraries (2 kb, 5 kb, 10 kb and 20 kb). FragScaff v. 140324 was further applied to build superscaffolds using the barcoded sequencing reads, generating a genome with a scaffold N50 size of 1080.3 Kb. The total length of this version is 1146.5 Mb.

**Table 3:** The *de novo* assembly statistics of *U. unicinctus* genome.

	Length		Number	
	Contig (bp)	Scaffold (bp)	Contig	Scaffold
Total	1,176,627,468	1,193,865,770	7,584	5,140
Max	4,054,850	5,933,956	—	—
Number >=2000	—	—	7,581	5,137
N50	540,731	1,106,205	586	294
N60	383,187	795,034	847	421
N70	258,812	572,589	1,221	598
N80	148,014	344,720	1,827	864
N90	64,976	130,407	3,055	1,421

### Hi-C library construction, sequencing and pseudo-chromosome anchoring

The Hi-C library was constructed by a standard protocol described previously with certain modifications^18^. Briefly, the mussel tissue of *U. unicinctus* was fixed with 1% formaldehyde solution in MS buffer (10 mM potassium phosphate, pH 7.0; 50 mM NaCl; 0.1 M sucrose), and the nuclei were enriched from flow-through and subsequently digested with HindIII restriction enzyme (NEB). Biotin-labeled DNAs were ligated and purified, followed by fragmenting to a size of 300–500 bp. After a quality control process, the constructed Hi-C library was sequenced on an Illumina HiSeq X Ten sequencer with paired-end 350 bp. In total, 159.47 Gb of high-quality Hi-C data with 132.89 × coverage was acquired (Table 1).

The clean Hi-C paired-end reads were assembled using ALLHIC v. 0.9.8^19^ containing five steps, namely pruning, partition, rescue, optimizing and building, with the following parameter settings: “allhic partition --pairsfile group.clean.pairs.txt --contigfile group.clean.countsGATC.txt -K 26 --minREs 50 --maxlinkdensity 3 --NonInformativeRabio 0”. Ultimately, the size of chromosome-level genome assembly is ~1113.8 Mb, of which 97.82% were anchored into 17 pseudo-chromosomes ranging from 41.8 Mb to 86.6 Mb in length (Fig. 1b and Table 4), containing 7,429 contigs with N50 of 531.5 kb and 1,394 scaffolds with N50 of 68.3 Mb (Table 5).

**Table 4:** The statistics of anchored rate and chromosome-level scaffold lengths.

Class	Scaffold Number	Total Length
Place	17	1,167,897,129
Unplace	1,601	25,968,641
Total	1,618	1,193,865,770
Anchored rate	97.82%	
**Sequence ID**	**Cluster Number**	**Sequence Length**
Chr1	247	60,918,862
Chr2	194	73,672,776
Chr3	173	77,575,773
Chr4	173	64,884,347
Chr5	161	65,285,633
Chr6	144	65,449,170
Chr7	170	49,572,668
Chr8	222	71,319,834
Chr9	223	81,417,580
Chr10	227	74,566,232
Chr11	261	62,915,995
Chr12	285	90,777,602
Chr13	252	79,577,786
Chr14	175	43,828,878
Chr15	223	74,974,652
Chr16	245	59,494,443
Chr17	238	71,664,898

**Table 5:** The assembly statistics for Hi-C.

Sample ID	Length		Number	
	Contig (bp)	Scaffold (bp)	Contig	Scaffold
Total	1,176,627,468	1,193,865,770	7,429	1,394
Max	4,054,850	90,777,602	—	—
Number>=2000	—	—	7,419	1,392
N50	544,229	71,664,898	583	8
N60	386,178	65,449,170	841	10
N70	264,043	64,884,347	1,208	12
N80	151,337	60,918,862	1,798	14
N90	66,330	59,494,443	2,989	15

### Annotation of repeats and non-coding RNA (ncRNA)

Homologous comparison and *de novo* prediction were applied to annotate the repeated sequences in the assembled genome. For homologous comparison, the RepeatMasker v. 4.0.7^20^ and the associated RepeatProteinMask v. 4.05^21^ were performed to align against Repbase database^22^. For *ab initio* prediction, LTR_FINDER v.1.07^23^, RepeatScout v. 1.05^24^ and RepeatModeler v. 1.05^25^ were first used for *de novo* candidate database constructing of repetitive elements. The repeated sequences were annotated using RepeatMasker v. 4.0.7 Tandem repeat sequences were *de novo* predicted using TRF v. 4.07b^26^. In total, 482.1 Mb repetitive sequences were annotated, accounting for 42.34% of the assembled *U. unicinctus* genome (Table 6). Among the repetitive sequences, DNA transposons (DNA), long interspersed elements (LINE), short interspersed nuclear elements (SINEs), and long terminal repeats (LTRs) accounted for 20.72%, 4.26%, 0.25%, and 10.60% of the whole genome, respectively (Table 7).

**Table 6:** The annotation of repeated sequences in *U. unicinctus* genome.

Type	Repeat Size(bp)	% of genome
Trf	109,563,172	9.18
Repeatmasker	465,606,698	39.00
Proteinmask	52,480,395	4.40
Total	505,521,819	42.34

**Table 7:** Summary statistics of repeat annotation in *U. unicinctus* assembly.

Type	Denovo + Repbase Length(bp)	% in Genome	TE proteins Length(bp)	% in Genome	Combined TEs Length(bp)	% in Genome
DNA	242,682,456	20.33	23,757,810	1.99	247,361,679	20.72
LINE	44,495,416	3.73	15,032,681	1.26	50,843,385	4.26
SINE	2,927,755	0.22	0	0	2,927,755	0.25
LTR	123,562,007	10.32	13,833,015	1.16	126,497,677	10.60
Other	1,067	0	0	0	1,067	0
Unknown	81,020,113	6.79	0	0	81,020,113	6.79
Total	465,606,698	39.00	52,480,395	4.40	472,502,097	39.58

For ncRNA annotation, the tRNAs were predicted using tRNAscan-SE v. 1.3.1 software^27^, and the rRNAs fragments were identified by searching against the Human rRNA database using BLAST with an E-value of 1E-10. Other ncRNAs, including microRNAs (miRNA) and small nuclear RNAs (snRNAs) were predicted by INFERNAL v. 1.1rc4^28^ using Rfam database^29^. Finally, a total of 5,908 ncRNAs were annotated, including 1,535 miRNAs, 3,431 tRNAs, 124 rRNAs, and 348 snRNAs in *U. unicinctus* genome(Table 8).

**Table 8:** Annotation of non-coding RNA genes in *U. unicinctus* assembly.

Type	Subtype	Copy(w)	Average length(bp)	Total length(bp)	% of genome
miRNA	—	1,535	127.2143322	195,274	0.016356
tRNA	—	3,431	76.69367531	263,136	0.022041
	rRNA	124	172.8225806	21,430	0.001795
	18 S	34	321.4705882	10,930	0.000916
rRNA	28 S	22	187.9090909	4,134	0.000346
	5.8 S	5	118.6	593	0.00005
	5 S	63	91.63492063	5,773	0.000484
	snRNA	348	156.3649425	54,415	0.004558
	CD-box	99	145.030303	14,358	0.001203
snRNA	HACA-box	28	242.7142857	6,796	0.000569
	splicing	219	150.6438356	32,991	0.002763

### Protein-coding gene prediction and function annotation

The structure of protein-coding genes were predicted by homology-based prediction, *de novo* prediction and transcriptome-based methods. For homologous annotation, the protein sequences of *Helobdella robusta* (GCA000326865.1), *Capitella teleta* (GCA000328365.1), *Lottia gigantea* (GCA000327385.1), *Crassostrea gigas* (GCA000297895.2), *Mizuhopecten yessoensis* (GCA002113885.2), *Octopus bimaculoides* (GCA001194135.2), *Drosophila melanogaster* (GCA000001215.4), *Anopheles gambiae* (GCA000005575.1), *Caenorhabditis elegans* (GCA004526295.1), *Mnemiopsis leidyi* (GCA000226015.1), *Nematostella vectensis* (GCA_932526225.1), *Trichoplax adhaerens* (GCA000150275.1), *Branchiostoma floridae* (GCA015852565.1), *Homo sapiens* (GCA000001405.29) were downloaded from the NCBI’s Genbank database, and aligned against *U. unicinctus* genome using TBLASTN v. 2.2.26^30^. The matching proteins were conjoined by Solar software v. 0.9.6^31^, and then aligned to homologous genome sequences for structural prediction by GeneWise v. 2.4.1^32^ (referred to “Homolog” in Table 9). Clean data of RNA-sequencing (RNA-seq) derived from intestines, blood, gonad, and muscle were assembled with Trinity (v2.0)^33^, and were then aligned against *U. unicinctus* genome using Program to Assemble Spliced Alignment (PASA)^34^ (referred to “PASA” in Table 9). Simultaneously, Augustus v. 3.2.3^35^, GeneID v. 1.4^36^, GeneScan^37^, GlimmerHMM v. 3.0.3^38^, and SNAP^39^ were employed for *ab initio* prediction, in which Augustus, SNAP, and GlimmerHMM were trained by homolog set gene models (referred to “*De novo*” in Table 9). Additionally, RNA-seq reads were directly mapped to *U. unicinctus* genome using Tophat v. 2.0.13^40^. The mapped reads were assembled into gene models (RNAseq-Cufflinks-set) by Cufflinks v. 2.1.1^41^ (referred to “Cufflinks” in Table 9). Finally, the gene models were integrated by EvidenceModeler v. 1.1.1^42^. We set the Weights for each type of evidence as follows: PASA-T-set > Homology-set > Cufflinks-set > Augustus > GeneID = SNAP = GlimmerHMM = GeneScan. In order to get the information of untranslated regions (UTRs) and alternatively spliced sites, PASA2 was used to update the final gene models (referred to “Pasa-update” in Table 9). In total, 21,524 protein-coding genes were predicted in the *U. unicinctus* genome with an average transcript and coding sequence (CDS) length of 5,391.7 bp and 1,291.94 bp, respectively (Table 9).

**Table 9:** The statistics of predicted protein-coding genes of *U. unicinctus* assembly.

Gene set	Category	Number	Average transcript length(bp)	Average CDS length(bp)	Average exons per gene	Average exon length(bp)	Average intron length(bp)
	Augustus	31,081	3,508.15	1,156.08	5.53	208.89	518.73
	GlimmerHMM	122,202	8,286.29	500.26	3.21	156	3,528.35
De novo	SNAP	60,568	3,228.72	747.58	4.12	181.27	794.22
	GeneID	29,364	23,668.70	632.2	4.87	129.89	5,957.13
	Genscan	56,190	12,648.12	1,051.14	5.14	204.48	2,800.77
	Cel	8,284	2,250.88	947.98	3.23	293.36	583.89
	Cgi	26,716	2,391.34	952.2	3.49	272.63	577.35
	Cte	55,334	1,657.30	699.91	2.7	259.4	563.76
	Dme	7,957	2,977.41	951.45	4.39	216.93	598.32
	Hro	31,197	1,356.93	561.38	2.29	244.69	614.68
	Hsa	12,701	2,944.95	940.47	4.34	216.92	600.94
Homolog	Lan	23,870	2,681.78	992	3.96	250.28	570.2
	Lgi	46,470	1,487.22	640.01	2.39	268.09	610.72
	Mye	35,576	2,222.15	890.57	2.93	303.55	688.55
	Obi	20,128	2,176.79	820.89	3.41	241	563.51
	Rpa	31,480	2,964.11	837.69	3.19	262.87	972.45
	Sma	23,994	1,765.84	720.3	2.77	260.11	590.96
	PASA	25,403	4,574.31	947.96	5.88	161.1	742.47
RNAseq	Cufflinks	40,192	6,143.97	1,446.11	4.78	302.63	1,243.33
	EVM	27,343	4,559.22	1,168.76	5.69	205.28	722.35
	Pasa-update*	27,178	4,599.60	1,182.99	5.77	205.03	716.31
	Final set	21,524	5391.7	1291.94	191.22	712.21	6.76

The symbol of ‘*’ indicated that the untranslated regions were included, and vice versa.

**Fig. 2:**
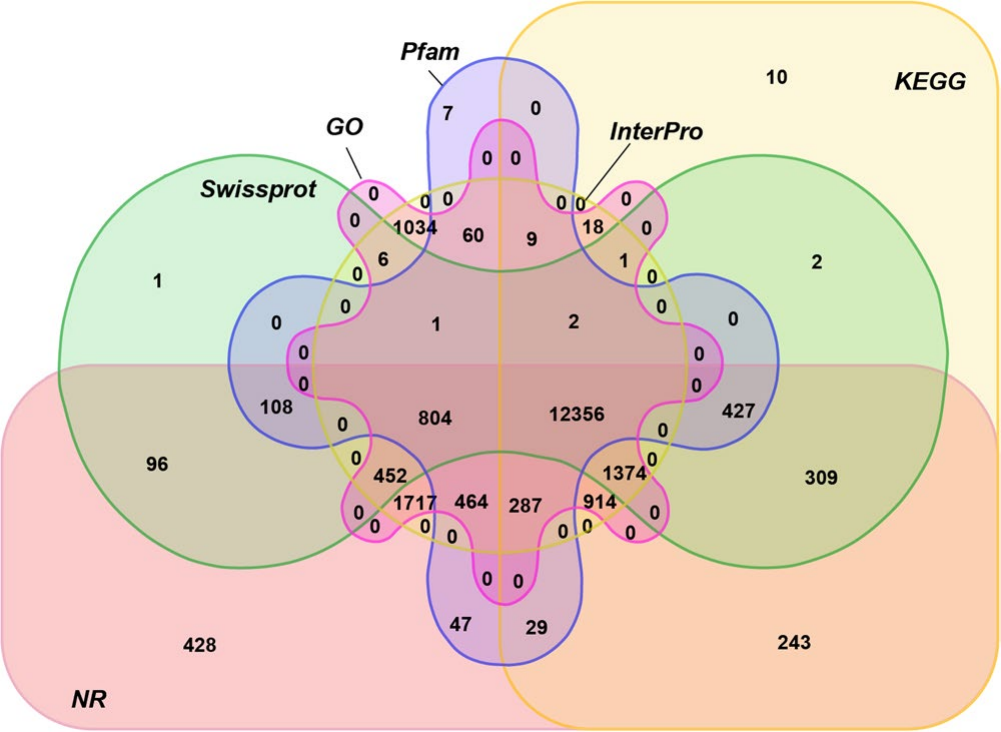
Venn diagram of functional annotation of the *U. unicinctus* protein-coding genes. The Venn diagram shows the shared and unique annotations among NR, KEGG, SwissProt, and InterPro.

For functional annotation, the predicted protein sequences were aligned against SwissProt^43^, NCBI’s non-redundant protein sequence databases (NR), InterPro^44^, Gene Ontology (GO)^45^, Kyoto Encyclopedia of Genes and Genomes (KEGG)^46^ and Pfam protein databases^47^ by BLASTP (E-value ≤ 1E-05) with the matched rates of 74.1%, 93.2%, 74.2%, 98.5%, 90.6%, and 67.8%, respectively (Table 10). InterproScan tool^48^ in coordination with InterPro database was applied to predict protein function based on the conserved protein domains and functional sites. In total, 21,408 genes were functionally annotated by at least one database, accounting for 99.5% of all predicted genes, among which 123,56 (68.33%) were supported by all six databases (Fig. 2).

**Table 10:** Functional annotation of the predicted protein-coding genes in *U. unicinctus*.

Type	Number	Percent(%)
Total	21,524	—
Swissprot	15,939	74.1
Nr	20,055	93.2
KEGG	15,981	74.2
InterPro	21,205	98.5
GO	19,499	90.6
Pfam	14,601	67.8
Annotated	21,408	99.5
Unannotated	116	0.5

## Data Records

All raw genomic sequencing data (Illumina, PacBio, Hi-C, 10× genomics) were deposited in the NCBI Sequence Read Archive (SRA) database with accession numbers SRR25893129^49^ and SRP458201^50^. Four transcriptome data from intestine, blood, gonad, and muscle were submitted to the NCBI SRA database with accession numbers SRR25683611, SRR25683610, SRR25683609, and SRR25683608, respectively, under accession the BioProject number PRJNA1006514^51^. The final chromosome assembly was deposited in the GenBank at the NCBI (JAXDRA000000000)^52^. The sequences of CDS, and protein and results of genome annotation, including repeat sequences, protein-coding regions, and ncRNA annotation, are available in figshare^53^.

## Technical Validation

### Quality validation of sequencing data

The quality control of Illumina, 10 × genomic and transcriptome sequencing data was assessed using FastQC quality control (http://www.bioinformatics.bbsrc.ac.uk/projects/fastqc/). The Q20 of Illumina sequencing data was greater than 95.92%, and Q30 was greater than 89.67%. The Q20 of 10× genomic sequencing data was greater than 92.71%, and Q30 was greater than 85.77%. The Q20 of transcriptome sequencing data was larger than 97.67%, and Q30 was larger than 93.41%. The low-quality reads (<Q20) were filtered to ensure the reliability of the data used in subsequent analyses.

### Assessment of the genome assembly

The quality of the genome assembly was assessed by four methods as follows: (i) The evaluation of the genome assembly by BUSCO v. 5.1.2^54^ suggested a high level of completeness (93.5%). Of 954 metazoa BUSCO genes, 92.7% were complete and single-copy, 0.8% were complete and duplicated, 4.0% were fragmented, and 2.5% were missing (Table 11). Additionally, completeness of the gene model prediction was also evaluated by BUSCO v. 5.1.2, generating a score of 91.6% (4.7% fragmented and 3.7% missing BUSCOs) (Table 12); (ii) Employing CEGMA (RRID: SCR 015055)^55^, we detected 240 (96.77%) of 248 core eukaryotic genes were detected in the genome assembly, including 221 (89.11%) complete genes. (iii) The clean short reads generated by the Illumina platform were mapped to the assembled *U. unicinctus* genome using BWA with parameters setting as ‘-o 1 -i 15’^56^. The results showed a mapping rate of 97.83% and a coverage rate of 93.90%; (iv) To evaluate the accuracy of the assembly at a single base level, variant calling with SAMTOOLS v. 0.1.19 was performed^57^. A total of 8,064,289 SNPs, including 7,985,055 heterozygous SNPs and 79,234 homozygous SNPs, were identified with a homozygous rate of 0.0081% (Table 13). All these results suggested the high completeness and accuracy of the *U. unicinctus* genome assembly.

**Table 11:** The BUSCO and CEGMA evaluation result of the genome assembly.

Type	BUSCO	CEGMA			
Number/ratio	C:93.5%[S:92.7%,D:0.8%] F:4.0%,M:2.5%,n:954	complete		complete + partial	
		# Prots	completeness	# Prots	completeness
		221	89.11%	240	96.77%

**Table 12:** The BUSCO score of the gene models.

Type	# Prots	Gene models % completeness
Complete BUSCOs (C)	873	91.6
Complete and single-copy BUSCOs (S)	861	90.3
Complete and duplicated BUSCOs (D)	12	1.3
Fragmented BUSCOs (F)	45	4.7
Missing BUSCOs (M)	36	3.7
Total BUSCO groups searched	954	100

**Table 13:** The statistics of SNP in *U. unicinctus* assembly.

Type	Number	Percentage (%)
All SNP	8,064,289	0.8254
Heterozygosis SNP	7,985,055	0.8172
Homology SNP	79,234	0.0081

## Data Availability

The software and pipelines used in this study were executed following the developers’ instructions, and the versions and parameters of these bioinformatic tools were described in the Methods section. If the parameter is not provided, the default value is used. No custom script or code was used.
